# Defective efferocytosis of vascular cells in heart disease

**DOI:** 10.3389/fcvm.2022.1031293

**Published:** 2022-09-30

**Authors:** Bandana Singh, Kathryn Li, Kui Cui, Qianman Peng, Douglas B. Cowan, Da-Zhi Wang, Kaifu Chen, Hong Chen

**Affiliations:** ^1^Vascular Biology Program, Department of Surgery, Harvard Medical School, Boston Children's Hospital, Boston, MA, United States; ^2^Center for Regenerative Medicine, University of South Florida Health Heart Institute, Morsani College of Medicine, University of South Florida, Tampa, FL, United States; ^3^Basic and Translational Research Division, Department of Cardiology, Boston Children's Hospital, Boston, MA, United States

**Keywords:** atherosclerotic cardiovascular disease, efferocytosis, myocardial infarction, macrophage—cell, atheroma, atherosclerotic plaque (AP)

## Abstract

The efficient phagocytic clearance of dying cells and apoptotic cells is one of the processes that is essential for the maintenance of physiologic tissue function and homeostasis, which is termed “efferocytosis.” Under normal conditions, “find me” and “eat me” signals are released by apoptotic cells to stimulate the engulfment and efferocytosis of apoptotic cells. In contrast, abnormal efferocytosis is related to chronic and non-resolving inflammatory diseases such as atherosclerosis. In the initial steps of atherosclerotic lesion development, monocyte-derived macrophages display efficient efferocytosis that restricts plaque progression; however, this capacity is reduced in more advanced lesions. Macrophage reprogramming as a result of the accumulation of apoptotic cells and augmented inflammation accounts for this diminishment of efferocytosis. Furthermore, defective efferocytosis plays an important role in necrotic core formation, which triggers plaque rupture and acute thrombotic cardiovascular events. Recent publications have focused on the essential role of macrophage efferocytosis in cardiac pathophysiology and have pointed toward new therapeutic strategies to modulate macrophage efferocytosis for cardiac tissue repair. In this review, we discuss the molecular and cellular mechanisms that regulate efferocytosis in vascular cells, including macrophages and other phagocytic cells and detail how efferocytosis-related molecules contribute to the maintenance of vascular hemostasis and how defective efferocytosis leads to the formation and progression of atherosclerotic plaques.

## Introduction

Efferocytosis or programmed cell death (PrCR) is an immunological non-inflammatory and evolutionarily-conserved program required for maintaining normal physiological function, development and tissue homeostasis by removing aged, damaged, and senescent cells (1, 2). The Greek-derived term “efferocytosis” refers to a tightly regulated process mainly involving the “eat me” and “don't eat me” molecules and related signaling pathways that drive phagocytic engulfment of apoptotic cells, but not off-target, healthy cells (3–5). The phagocytosis of apoptotic cells is maintained by both professional phagocytes such as macrophages, immature dendritic cells, and non-professional phagocytic cells (e.g., neighboring smooth muscle cells and endothelial cells). Macrophages, the main phagocytic cell type, play an important role in identifying dying cells for subsequent phagocytosis and clearance that would otherwise become intolerant of self-antigens and induce secondary necrosis. Moreover, the importance of efferocytosis in tissue hemostasis in physiological conditions is widely appreciated. Defective efferocytosis is believed to be an important feature of various autoimmune and chronic inflammatory diseases such as rheumatoid arthritis, atherosclerosis and systemic lupus erythematous (6). The treatment of efferocytosis-related disease has not yet been rectified. In this review, we summarize the underlying regulatory pathways of defective efferocytosis in the progression of cardiovascular disease and focus on future translational studies. Exploring the signaling molecules and regulatory molecular mechanisms associated with impaired efferocytosis in advanced atherosclerosis should enhance our knowledge for developing anti-atherosclerotic therapies focusing on improving efferocytosis.

## Basic steps of efferocytosis

Efferocytosis is described as a highly-conserved, programmed cell removal process involving synergistic regulation of the engulfment and removal of apoptotic cells *via* phagocytes. Effective efferocytosis requires the accurate recognition, phagocytosis, and removal of apoptotic cells. Efferocytosis is regulated through several signaling molecules: (a) “find-me” signals: different chemokines, nucleotides, other proteins, lipid and lipid products released from apoptotic cells that recruit phagocytes to the area of cell death; (b) “bridging molecules” signals: opsonin like molecules that connect phagocytes to their targeting apoptotic cells; (c) “eat me” signals: cell surface ligand molecules that recognize and bind to the engulfment receptor on the phagocytes through bridging molecules and that initiate efferocytosis; (d) “don't eat me” signals: molecules such as CD47, which is ubiquitously expressed on viable cells that separate them from apoptotic cells and inhibit phagocytosis. These signal molecules regulate the efferocytotic processes and determine whether a cell is denoted for engulfment and removal from the body or ignored by phagocytic cells (7, 8).

## “Find-me” signals in efferocytosis

Studies show that in the region of cell death, apoptotic cells release various molecules carrying “find-me” signals including nucleotides ATP, UTP (9), lysophosphatidylcholine (LPC) (10), sphingosine 1- phosphate (11), and CX_3_C-chemokine ligand 1(CX_3_CL1) (12). Some “find me” molecules express different signaling peptides that are required for preparing the microenvironment for cell clearance (13). Macrophages are guided by “find me” signals and rapidly migrate to the area of cell death for removing apoptotic corpses (7). Then, macrophages bind either directly or indirectly to the “eat- me” signal expressed on the surface of the apoptotic cells through “bridging molecules” (14).

## “Eat-me” signals in efferocytosis

While several “eat-me” signals have been identified, mainly phosphatidylserine (PtdSer), intercellular adhesion molecules 3 (ICAM3), and calreticulin (Calr), these are required for the phagocytosis of apoptotic cells (15, 16). Among of them, PtdSer is the main “eat-me” signal. Under physiological conditions, PtdSer is located on the inner surface of the plasma membrane, but PtdSer in dying cells is reverted to the outer surface of the plasma membrane where it binds to the receptor of the phagocyte (17).

## “Bridging-molecules” in efferocytosis

Upon arrival of the macrophage to the area of cell death, the macrophage directly binds to extracellular membrane-bound PtdSer through stabilin 1, stabilin 2, T cell immunoglobulin mucin receptors TIM1, TIM3, TIM4, or through GPCR brain angiogenesis inhibitor 1 (BAI1) (18–21). In some cases, macrophages bind to several bridging molecules, such as Gas6 and protein S, that bind to the tyrosine kinase receptor (TAM), to facilitate the interaction with PtdSer. In other cases, thrombospondin or MFG-E8 binds both PtdSer and integrins αVβ3 and αVβ5 or CD36. In addition, PtdSer-related receptors have different characteristics; some of the receptors (MerTK, BAI1 and integrins) play a role in the signaling process and others (e.g., Tyrosine kinase receptor and CD36) play a role in tethering and adhesion.

## Phagocytosis of dying cells

The PtdSer on apoptotic cells binds to the PtdSer receptor on macrophages that form a phagocytic cup through actin cytoskeleton remodeling and the formation of filamentous (F)-actin around the apoptotic cell, promoting internalization of apoptotic cells into the phagosome and mechanical retraction of the phagosome into cells (22–24). The activated small GTPase family members (i.e., Rac1, Cdc42, and RhoA) are involved in the formation of the phagocytic cup and the internalization of the phagosome (25). The effector of Rac1 activation also regulates the internalization of apoptotic cells through the association of adaptor proteins with the Rac GEF DOCK180 to activate Rac1 and initiate phagocytic cup formation, and leads to phagocytosis (26). Membrane trafficking is also important for efferocytosis, like the cytoskeleton remodeling that underlies Drp1-dependent mitochondrial fission. Mitochondrial fission increases cytosolic calcium by releasing endoplasmic reticulum calcium into the cytosol that drives vesicular trafficking.

After internalization of apoptotic cells, an autophagy-related protein LC3 binds to phagosomal membrane lipids through LC3-associated phagocytosis (LAP) and promotes lysosome degradation of apoptotic cell constituents (27). After phagolysosomal degradation of apoptotic cells, these macromolecular constituents are loaded into macrophages, then macrophages can either use or efflux these constituents through specific mechanisms. As a result, cholesterol released from degraded apoptotic cell induces the expression of ABCA1 and ABCG1 through activating peroxisome proliferate-activate receptor (PPAR) and liver X receptor (LXR) and leading to cholesterol efflux from the cells (28). Macrophage lysosome contains DNase II that degrades chromosomal DNA derived from degraded apoptotic cells. It has been reported that mice lacking DNase II induce an autoimmune disease, polyarthritis, similar to rheumatoid arthritis in humans (29).

## Defective efferocytosis in heart disease

Coronary heart disease stems from atherogenesis and plaque vulnerability has been associated with the accumulation of apoptotic and necrotic debris (30–33). A necrotic core contributes to plaque expansion that disrupts luminal flow and, in turn, reduces coronary perfusion, leading to detrimental heart diseases such as ischemic myocardial infarction. Many studies report that impaired efferocytosis induces some changes in blood flow, which is directly related to plaque vulnerability and atherogenesis (34). In mammalian cells, efferocytosis avoids intracellular accumulation of membrane-derived lipids by initiating the reverse cholesterol transport (RCT) machinery. In normal situations, apoptotic cells express externalized phosphatidylserine (PS) that upregulates ABCA1 in macrophages, which, in turn, induces the efflux of cholesterol to ApoA1 (35, 36). The necrotic core also expresses PS, but it fails to show similar responses for efflux of cholesterol to ApoA1 (35). Furthermore, in defective efferocytosis conditions, the signals that initiate the reverse cholesterol transport pathway in vascular cells are suppressed, leading to the formation of foam cells and the initiation of atherosclerosis.

**Figure 1:**
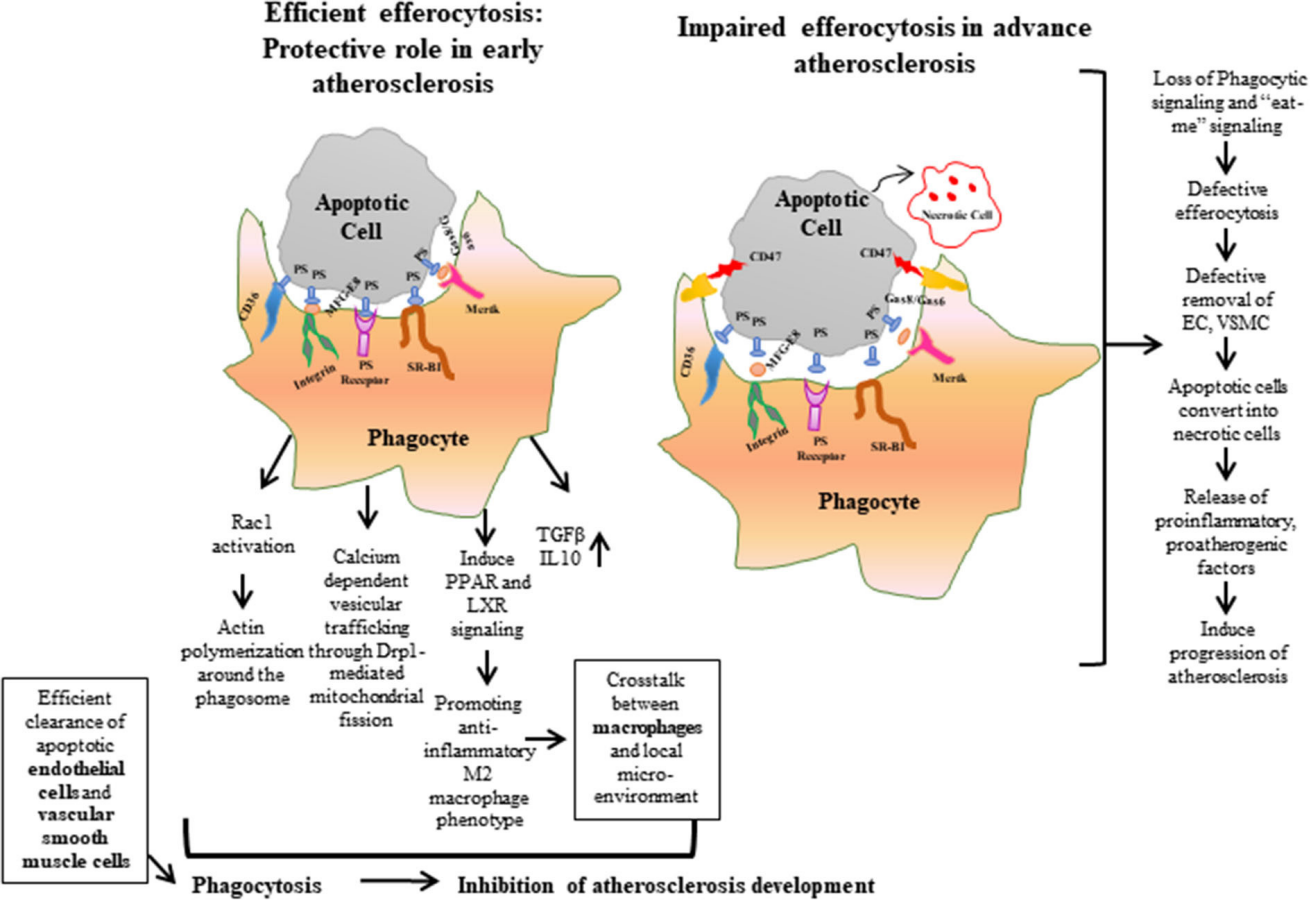
Efferocytosis is the phagocytic process required for maintaining normal vascular hemostasis. Efficient efferocytosis plays an important role in protecting against atherosclerosis by inducing phagocytic signals or “eat-me” signals and clearing apoptotic debris. Conversely, in defective efferocytosis, the phagocytic signals are reduced and the phagocytic ability of apoptotic cells is decreased. As a result, apoptotic cells are converted to necrotic cells, and accumulation of these uncleared cells form a necrotic core, which leads to the release of proatherogenic factors and progression of atherosclerosis plaque. ECs, Endothelial cells; VSMCs, Vascular Smooth Muscle Cells.

In normal physiological conditions, efferocytosis suppresses inflammation by preventing accumulation of toxic cellular contents. Macrophages release interleukin (IL)-10 and transforming growth factor (TGF)-β to help clear dying cells and induce anti-inflammatory signaling (37–39). But, when efferocytosis fails to remove apoptotic debris, phagocytic cells convert to inflammatory cells, which leads to non-resolving vascular inflammation (36, 40, 41). Impaired efferocytosis leads to rapid degradation of apoptotic cell membranes, and, consequently, secretion of intracellular content to the interstitium (Figure 1). These intracellular materials contain cytokines and proteases that destabilize the plaque and promote angiogenesis of the vascular cells in the plaque, respectively, as well as the release of thrombogenic factors that play a role in atherogenesis and promote plaque vulnerability (32, 42). Thus, impaired efferocytosis can be viewed as a defective waste management program that plays a key role in the vascular biology of atherogenesis. Taken together, defective efferocytosis stimulates release of cytokines that promote plaque inflammation, and impairs reverse cholesterol transport that promotes foam cell accumulation and also induces plaque vulnerability through atherothrombotic modification in the extracellular matrix, which leads to pathogenesis and progression of atherosclerosis.

## Mechanisms behind impaired efferocytosis in atherosclerosis

### Reduced apoptosis

The accumulation of apoptotic cells and expansion of the necrotic core associated with atherogenesis, in turn, restricts luminal flow and reduces coronary perfusion (30–33). Studies have shown that impaired efferocytosis induces other maladaptive factors that directly cause atherogenesis and plaque vulnerability (34). Defective efferocytosis involved in lipid accumulation and secondary necrosis causes inflammatory responses and autoimmune responses. Studies have demonstrated that when high capacity efferocytosis occurs in early lesions (i.e., efferocytosis works properly), there is no accumulation of apoptotic cells (43, 44). It has been reported that reduction of the absolute number of phagocytes leads to weakened phagocytic ability of phagocytes.

During the progression of atherosclerosis, endothelial dysfunction and ER stress leads to macrophage and VSMC apoptosis and reduced phagocytic capacity (45). A reduced M2 macrophage population, increases macrophage polarization toward pro-inflammatory M1 phenotype, and lessens the phagocytic ability of smooth muscle cells in atherosclerosis (46, 47). Studies also show that reductions of phagocytic receptors on macrophages (i.e., CD36, Mertk and LRP1) resulted in a loss of their ability to phagocytically clear apoptotic debris. Increased expression of metalloproteinase, disintegrin and ADAM17 were also found in atherosclerotic plaques that cause reduced expression of Mertk and LRP1 on macrophages (48, 49). The inhibition of protein kinase B activation leading to decreased expression of LRP1 on macrophages causes plaque growth during atherosclerotic lesion progression (50). LRP1 receptor deficiency induces the secretion of pro-inflammatory cytokines such as TNF-α, monocyte chemoattractant protein-1 (MCP-1), and MMP-9 which causes reduced efferocytosis. Notably, it has recently been reported that the endocytic adaptor proteins known as epsins target ubiquitin-dependent internalization and downregulation of LRP1 in macrophages in hyperlipidemic conditions, hindering effective efferocytosis in macrophages and propelling atherosclerosis progression (51–53). Interestingly, it has been shown that deletion of LRP1 induces CCR7 expression in M1 macrophages and promotes atherosclerosis regression (54).

### Endothelial dysfunction and macrophages in the pathophysiology of atherosclerosis

Atherosclerosis is a chronic inflammatory disease and common cause of cardiovascular disease (CVD), characterized by the thickening of the intima of large and medium-sized arteries (55). Abnormal immune responses, resulting from defective lipid metabolism leads to the accumulation of modified lipoproteins beneath the endothelium, inducing the formation of lipid rich plaques or “atheromas.” The accumulation of apoptotic cells plays an important role in atherosclerotic progression and plaque stability (56–58). Although, how high cholesterol concentrations leads to the development of atherosclerosis remains unclear, it is believed that higher blood cholesterol levels is a common cause for the pathogenesis of atherosclerosis (57).

Excessive LDL-C forms reactive oxygen species in the intima and promotes the formation of foam cells by binding the LRP receptor on vascular phagocytes. As atherosclerosis disease progresses, foam cells fail to modify the lipoprotein and fail to distinguish the destructive lipoprotein, which leads to apoptosis by inducing endoplasmic reticulum stress and ROS production (45). In addition, the areas of disturbed laminar flow in the arterial tree are more prone to lipoprotein accumulation as well as plaque formation. Over time, the rupture of foam cells leads to thrombus formation overlying the plaque and the occlusion of coronary vessels in the heart, leading to episodes like myocardial infarction and stroke (56).

The healthy endothelium plays a protective role against plaque formation through increased nitric oxide bioavailability, decreased adhesion molecule expression, and increased anti-inflammatory signaling process (59). Endothelial dysfunction (i.e., damaged endothelium) upregulates the expression of cell surface adhesion molecules which promote infiltration of macrophages and T lymphocytes into the atherosclerotic plaque (60, 61). Additionally, endothelial cells take part in cholesterol transcytosis through increasing expression of scavenger receptors that bind to the modified lipoproteins and transport them across the endothelium into arterial intima (62, 63). Accumulation of lipoproteins in the intima leads to endothelial activation, which induces sterile inflammation and further modification of retained lipoproteins in the plaque (56, 63).

However, many risk factors take part in inflammatory activation and a great deal of research has demonstrated that macrophages play key roles in the pathogenesis of atherosclerosis by sustaining a continuous inflammatory state and through the secretion of inflammatory mediators such as cytokines and chemokines. Macrophages also take part in the efferocytotic process. In the early stage of atherosclerosis, macrophages play a role in the efferocytotic clearance of apoptotic debris, which differs from lipid-laden foam cells. In the late stages of atherogenesis, macrophages produce pro-inflammatory mediators (60, 64, 65). For example, macrophages release major pathological proinflammatory cytokines (e.g., IL-6, IL-β, and TNFα) that play important roles in atherosclerotic plaque progression (58). Moreover, M2 macrophage populations elicit greater efferocytosis ability than M1 macrophage populations (66).

In murine models of atherosclerosis, higher levels of circulating monocytes have been found, supporting the idea that atherosclerosis not only affects vasculature, but also has a systemic impact on hematopoiesis (67, 68). Activated endothelial cells express some cell surface adhesion molecules, such as P- and E- selectin, that bind with their respective receptors expressed on the cell surface of monocytes, and stimulate monocyte rolling (69, 70). The tethering and transmigration of monocytes to the intima is followed by monocyte differentiation into macrophages in intima through binding of monocyte integrin very late antigen-4 (VLA-4) and lymphocyte function associated antigen (LFA-1) with their respective ligands—vascular adhesion molecule-1 (VCAM-1) and intracellular adhesion molecule-1 (ICAM-1) on the activated endothelium (37, 60).

In the initial stage of atherosclerosis, macrophages limit the expansion of early atheroma through efferocytotic clearance of apoptotic cells and debris (44, 71). In advance stages of atherosclerosis, macrophages play a role in development of necrotic cores and the thinning of fibrous caps (72). Macrophages contribute to fibrous cap thinning through two different mechanisms. One way is by inducing vascular smooth muscle cell apoptosis through involving Fas death receptor and production of pro-inflammatory, apoptotic cytokines (i.e., TNF-α) (73). Macrophages can also produce matrix metalloproteinases (MMP) that lead to the degradation of collagen which in turn destabilizes the cap (72). Specifically, MMP2 and MMP9 are involved in macrophage mediated fibrous cap thinning (74). Macrophages are also involved in the destabilization of atherosclerotic plaques which leads to the generation of necrotic cores (75, 76). The apoptosis of residential macrophages in the intima, along with the impaired efferocytosis of apoptotic cells from surrounding macrophages, induces the generation of a necrotic core and leads to the pathogenesis of atherosclerotic plaque progression (75, 76).

### Upregulation of “don't eat-me” signals

It has been shown that the increased levels of proinflammatory molecule TNF-α in atherosclerotic tissues upregulates CD47, an important “don't eat me” molecule in the atherosclerosis plaque. Additionally, studies have shown that atherosclerotic mouse models treated with CD47 blocking antibodies improved atherosclerosis by enhancing clearance of dead vascular tissue and reversing impaired efferocytosis (77). It has been reported that a conserved mammalian lncRNA, myocardial infraction–associated transcript (MIAT), upregulates the expression of CD47 by sponging miR-149-5p and shows higher expression in atherosclerosis patients (78). High mobility group box1 protein (HMGB1), a pro-inflammatory molecule, inhibits phagocytosis by binding to PS expressed on the surface of apoptotic neutrophils. Consequently, pretreatment of macrophages with HMGB1 blocked efferocytosis as a result of the diminished activity of MFG-E8 factor, which bridges PS and integrin on the surface of phagocytes (79).

### ER stress and ROS production

During the progression of atherosclerosis, ER stress leads to ROS production and oxidation of LDL. It has been reported that high density lipoprotein (HDL) upregulates the expression of SR-BI receptors on phagocytes which inhibit the ox-LDL induced free cholesterol accumulation and ER stress that impairs efferocytosis (80). Ox-LDL upregulates the expression of toll-like receptor-4 (TLR-4) reduces the expression of SR-BI and LRP1. This reduced expression ultimately leads to an increase in the secretion of pro-inflammatory cytokines TNF-α and IL-1β, which in turn inhibit the activation of liver X receptor and reduce apoptotic clearance (81).

Studies have shown that defective efferocytosis is related to impaired macrophage phagocytosis. Transcription factor interferon regulatory factors (IRF5) play important roles in modulation of myeloid functions and programming. IRF5 also regulates efferocytosis and necrotic core formation in the atherosclerotic lesion. It has been reported that transcriptional regulator interferon regulatory factor 5 (IRF5) modulates the expression of proinflammatory CD11c+ macrophage phenotype within the atherosclerotic lesion and impairs efferocytosis by suppressing the expression of integrin receptor MFGE8 and Itgb3 (82). Furthermore, studies have shown that loss of IRF5 reduced the expression of CD11c^+^ inflammatory macrophage phenotype within the atherosclerotic lesion. Deficiency of IRF5 increases the efferocytosis by CD11c^−^ macrophages through increased expression of integrin β-3 (Itgb3) and milk fat globule-epidermal growth factor 8 (Mfge8) proteins (82). The inhibition of recognition of apoptotic cells by phagocytes in the atherosclerotic plaque is one of the reasons why atherosclerotic plaques exhibit defective efferocytosis.

### Epigenetic modification

It has been also shown that dysfunctional microRNAs (miRs), a type of non-coding RNA, are associated with post-transcriptional modifications of gene expression that causes defective efferocytosis. Studies have shown that in early lesions, miR-155 plays an important role in impaired efferocytosis and macrophage proliferation through the targeting of colony-stimulating factor-1 receptor. In advanced stages of atherosclerosis, miR-155 suppresses the expression of B-cell leukemia/lymphoma 6 (Bcl6) and accelerates foam cell accumulation in the atherosclerotic lesion (83). Bcl6, a potent transcriptional inhibitor, decreases RhoA activity, modulates cytoskeletal remodeling of macrophages and impairs efferocytosis (84).

Lastly, a genome-wide association study of coronary atherosclerosis patients reveals that the 9p21.3 allele variant was related to atherosclerosis lesion burden and impaired efferocytosis. A GWAS study has also shown that the 9p21.3 locus is associated with a reduced expression of cyclin-dependent kinase inhibitor 2B (CDKN2B) and “eat me” ligand calreticulin (Calr). This leads to defect efferocytosis which is unable to remove large numbers of apoptotic vascular smooth muscle cells and causes the expansion of the atherosclerotic plaque (36, 85).

## Conclusions

Under normal conditions, the phagocytic capacity of macrophages is sufficient to remove apoptotic cells completely. The reduction of phagocytic capacity and factors that inhibit the clearance of diseased vascular cells, such as genetics and inflammation, play an imperative role in the pathophysiology of atherosclerotic efferocytosis—a role that is worthy of future translational research.

Myriad research studies have been conducted in an effort to better understand the underlying causes of atherosclerosis. Emerging evidence suggests that the impairment of efferocytosis is a root cause of atherosclerosis and plaque vulnerability over the time. Thus, therapies targeting efferocytosis will provide a new platform for the treatment and prevention of cardiovascular disease through the limiting the necrotic core. Encouragingly, it has been shown that these therapeutic agents are safe and specific in ongoing clinical trials.

## Author contributions

BS, KL, and HC wrote the manuscript. BS created figures. All authors performed the literature search and approved the final version of the manuscript.

## Funding

This work was supported in part by NIH grants R01HL093242, R01HL146134, R01HL137229, R01HL156362, R01HL16236, and R01HL158097 to HC, R01HL130845 and R01HL141853 to D-ZW, and American Heart Association Transformative Program Award to HC.

## Conflict of interest

The authors declare that the research was conducted in the absence of any commercial or financial relationships that could be construed as a potential conflict of interest.

## Publisher's note

All claims expressed in this article are solely those of the authors and do not necessarily represent those of their affiliated organizations, or those of the publisher, the editors and the reviewers. Any product that may be evaluated in this article, or claim that may be made by its manufacturer, is not guaranteed or endorsed by the publisher.
